# The Potential of a Robot Presence in Close Relationship to Influence Human Responses to Experimental Pain

**DOI:** 10.3390/life15020229

**Published:** 2025-02-04

**Authors:** Aya Nakae, Hani M. Bu-Omer, Wei-Chuan Chang, Chie Kishimoto, Yuya Onishi, Hidenobu Sumioka, Masahiro Shiomi

**Affiliations:** 1 Presence Media Research Group, Hiroshi Ishiguro Laboratories, Deep Interaction Laboratory Group, Advanced Telecommunications Research Institute International (ATR), 2-2-2 Hikaridai, Seika-Cho, Soraku-Gun, Kyoto 619-0288, Japan; hbuomer@atr.jp (H.M.B.-O.); chou@atr.jp (W.-C.C.); kishimotochie@atr.jp (C.K.); sumioka@atr.jp (H.S.); 2 Global Center for Medical Engineering and Informatics, Graduate School of Medicine, Osaka University, 2-2 Yamadaoka, Osaka 565-0871, Japan; 3 Laboratory of Science & Innovation for Pain, Graduate School of Frontier Biosciences, Osaka University, 2-2 Yamadaoka, Osaka 565-0871, Japan; 4 Department of Interaction Science Laboratories, Deep Interaction Laboratory Group, Advanced Telecommunications Research Institute International (ATR), 2-2-2 Hikaridai, Seika-Cho, Soraku-Gun, Kyoto 619-0288, Japan; y-onishi@atr.jp (Y.O.); m-shiomi@atr.jp (M.S.)

**Keywords:** communication robots, human–robot interaction, robotic healthcare applications, hormonal responses, experimental pain, pain management, mood enhancement

## Abstract

Pain management is a critical challenge in healthcare, often exacerbated by loneliness and emotional distress. This study investigated the potential of a communication robot, Moffuly, to reduce pain perception and influence hormonal responses in a controlled experimental setting. Nineteen healthy participants underwent heat pain stimulation under two conditions: with and without robotic interaction. Pain levels were assessed using the Short-form McGill Pain Questionnaire and the Visual Analogue Scale, while mood and mental states were evaluated through established questionnaires including the Profile of Mood States, Hospital Anxiety and Depression Scale, and Self-Rating Depression Scale. Hormonal changes, including cortisol, growth hormone, oxytocin, estradiol, and dehydroepiandrosterone-sulfate, were measured from blood samples collected at key time points. The results demonstrated significant reductions in subjective pain and improvements in mood following robotic interaction. These effects were accompanied by favorable hormonal changes, including increased oxytocin and decreased cortisol and growth hormone levels. The findings suggest that robotic interaction may serve as an innovative approach to pain management by addressing both physiological and psychological factors. This study highlights the potential of robotics to complement traditional therapies in alleviating pain and enhancing emotional well-being. By mitigating emotional distress and loneliness, robotic interventions may enhance existing pain therapies and offer innovative solutions for resource-limited healthcare systems.

## 1. Introduction

Pain is a complex and often persistent challenge in healthcare, and its treatment frequently falls short of providing adequate relief [1,2,3]. In the USA, the opioid crisis—arising from the overprescription of opioids for long-term non-cancer pain—has underscored the urgent need for alternative, comprehensive care strategies [4,5]. While the impact has been less pronounced in Europe, this global issue highlights the necessity of exploring innovative approaches to pain management. Although these comprehensive care strategies are beneficial, their implementation is often constrained by workforce limitations, particularly as aging populations increase the demand for healthcare services. This growing challenge underscores the need for innovative solutions, including the integration of robotic systems to complement human efforts in delivering effective pain management.

There exists a strong connection between loneliness and pain; individuals experiencing loneliness are more susceptible to chronic pain, while those suffering from pain tend to feel isolated [6]. Approximately 20% of elderly individuals suffer from loneliness related to pain [7]. Traditionally, addressing loneliness requires human intervention, but humanoid robots have shown potential in alleviating negative emotions, with several research protocols publicly available [8]. Emerging technologies may enable robots to perform tasks typically carried out by humans [9].

Cultural differences play a critical role in shaping attitudes toward human–robot interactions. Factors such as trust, acceptance, and willingness to engage with robots can vary significantly across cultural contexts. For example, societies with collectivist values may view robots as collaborative tools for fostering social bonds, while individualistic cultures might prioritize their practical utility [10,11,12]. Although globalization has reduced certain cultural barriers, nuances in robot acceptance and interactions remain significant [13].

Engaging with robots can have a relaxing effect on individuals by influencing their cortisol levels [14]. Robots designed for human interaction, known as communication robots, can help reduce loneliness by serving as perceived companions. Research has demonstrated that companion robots can effectively decrease feelings of loneliness from a psychological standpoint [15,16,17].

The sympathetic nervous system is activated by pain, resulting in the release of cortisol. Studies have shown that individuals with chronic pain exhibit diverse cortisol responses to experimental pain stimuli [18]. Many survivors of abuse report ongoing pain issues related to growth abnormalities, suggesting a connection to growth hormone (GH) production. GH plays a role in cognitive function [19] and pain perception, with disruptions in the GH/IGF-1/ghrelin system associated with increased pain sensitivity and low GH levels linked to heightened pain responsiveness [20].

Animal research has demonstrated that oxytocin (OT), a neuropeptide produced in the hypothalamus, influences pain processing. Some human studies have explored pain relief by targeting socioemotional aspects [21,22,23]. Estrogen, the primary female sex hormone, affects pain, along with other symptoms [24]. A lack of estrogen can lead to depressive moods, which, in turn, can increase sensitivity to pain [25].

This research sought to investigate whether interactions with a communication robot could modify pain perception under experimental conditions. Additionally, it aimed to explore hormonal changes in response to pain stimuli and how these fluctuations might be affected by robot intervention, utilizing various questionnaires and subjective pain assessments.

## 2. Methods

This study was approved by the Ethics Committee of the Osaka University Graduate School of Frontier Biosciences (Approval Number: FBS2020-13) in accordance with the tenets of the Declaration of Helsinki and was conducted between 29 July 2021 and 30 September 2021. The study was registered on the Japan Registry of Clinical Trials (jRCT ID: jRCT1090220290; first registered on 28 April 2017 and last modified on 20 October 2021). All participants provided written consent before enrollment in the study and were informed of their right to withdraw from the study at any time without penalty.

### 2.1. Study Design

**Figure 1:**
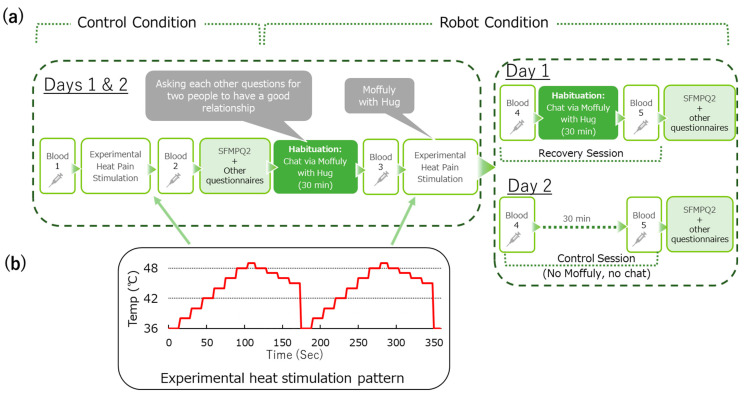
Study design: (**a**) study protocol; (**b**) experimental heat stimulation pattern.

This study employed a crossover design to examine the effects of robotic interaction on pain perception, hormonal changes, and psychological states during experimental heat pain stimulation. Participants attended two experimental sessions across two days, each consisting of a Control condition and a Robot condition, as illustrated in Figure 1a. The sequence of sessions was counterbalanced across participants to minimize order effects. Randomization was performed using a random number generator to assign participants to start with either the Day 1 or Day 2 session. Blinding was not feasible due to the nature of the robotic intervention; however, all data collection, including pain assessments, questionnaires, and blood sampling, was performed by the same trained investigators to ensure consistency and minimize variability.

**Figure 2:**
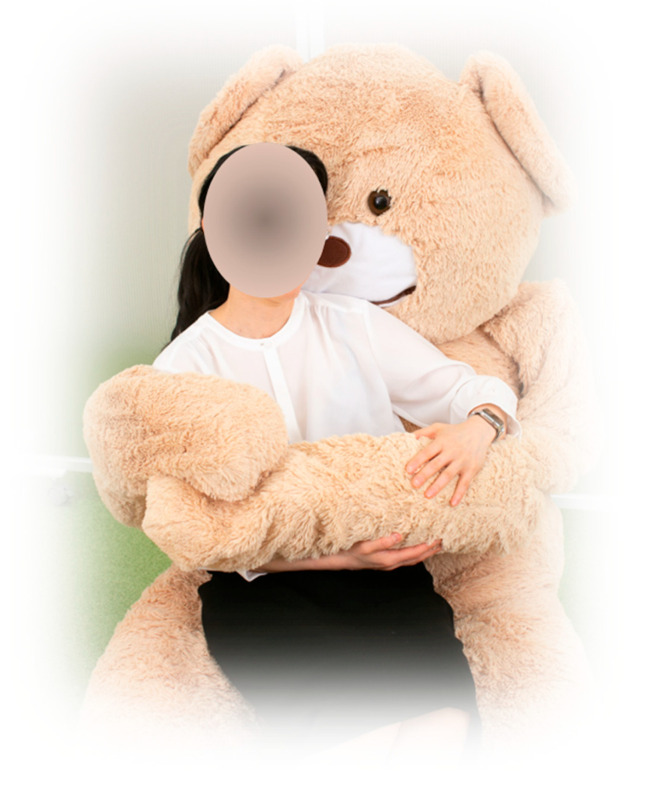
Moffuly robot used in the study.

The communication robot used in this study, Moffuly, is shown in Figure 2. Moffuly is a modified robotic bear designed to perform comforting behaviors, including preprogrammed hugging [26]. The robot was chosen for its ability to perform these comforting behaviors, with a particular focus on its hugging action, which was hypothesized to influence pain perception and hormonal responses. While Moffuly is also capable of engaging in conversations, this component was included during the familiarization phase to reduce novelty effects and ensure participant comfort with the robot. The robot’s human-like size and design were secondary factors intended to enhance the sense of a social presence, further supporting the study’s goal of evaluating the effects of physical and emotional interactions on pain alleviation.

During the Robot condition, Moffuly was operated remotely by an operator using a simple control interface to initiate the robot’s actions. For example, when the operator pressed a button, the robot closed its arms, hugged the participant briefly, and then opened its arms again.

#### 2.1.1. Control Condition

Each session began with heat pain stimulation, followed by the completion of questionnaires, including the Japanese version of the Short-Form McGill Pain Questionnaire (SF-MPQ-2), Profile of Mood States 2nd Edition (POMS-2), and Hospital Anxiety and Depression Scale (HADS).

#### 2.1.2. Robot Condition

To familiarize participants with Moffuly, a 30-min habituation session was conducted using an audio system embedded around the robot’s face. During this session, participants engaged in a conversation guided by 15 predefined questions (listed in Table 1). The conversational counterparts were of the opposite sex, unfamiliar to the participants, and varied between conditions to avoid bias.

Following the habituation session, participants underwent thermal heat pain stimulation while Moffuly provided a comforting hug throughout the stimulus duration.

Moffuly’s actions, such as opening and closing its arms to perform a hug, were remotely controlled by a human operator located in another room. The operator triggered these actions using a simple control interface to ensure precise timing. Additionally, the operator acted as the conversational counterpart during the habituation session, speaking through the robot’s audio system. While the operator’s presence was necessary to manage these robot behaviors, their role was limited to controlling the robot and facilitating the conversation. Participants had no direct interaction with or awareness of the operator’s presence.

The two robot sessions differed only in their concluding activities:

- Day 1: The Robot condition concluded with a Recovery Session, during which participants casually chatted with Moffuly for 30 min before providing the final blood sample and completing the questionnaires.
- Day 2: The Robot condition concluded with a Control Session, during which participants remained seated and relaxed for 30 min before providing the final blood sample and completing the questionnaires.

Blood samples were collected at the following specific time points during both conditions to measure hormonal changes: before and after each heat pain stimulation and at the end of each session (after the Recovery or Control Session), as depicted in Figure 1a.

### 2.2. Participants

Nineteen healthy participants (thirteen females and six males, aged 19−66 years, mean age: 39) were recruited through Osaka University’s intranet and a human resources company. Inclusion criteria required participants to be in good physical and mental health, with no history of chronic pain or ongoing medical conditions. Exclusion criteria included any medical history related to chronic pain disorders, neurological conditions, or use of medications affecting hormonal levels. All participants provided written informed consent before enrollment in the study and were informed of their right to withdraw from the study at any time without penalty.

The sample size was determined based on practical feasibility and prior studies with similar experimental designs in pain research and human–robot interaction [14,27,28,29]. Recruitment involved intensive data collection, including hormonal assays, pain sensitivity evaluations, and robotic interactions, which required careful coordination and limited participant availability. Despite these constraints, the sample size was sufficient to observe significant trends, providing a valuable foundation for future research.

Under the Robot condition shown in Figure 1a, participants were randomly assigned to start with either Day 1 or Day 2 (Recovery or Control session); half of the participants started with the Recovery session, while the other half started with the Control session. All participants attended the Recovery session, whereas 3 of the 19 participants dropped out before completing the Control condition. The data from all participants who completed both sessions were included in the statistical analyses.

### 2.3. Pain Stimulation and Measures

#### 2.3.1. Experimental Pain Stimulation and Subjective Assessment of Pain

Heat stimulation was chosen as the pain-inducing factor in this study due to its reproducibility, controlled delivery, and widespread use in pain research [30,31]. Unlike mechanical or electrical stimuli, heat pain allows for the consistent activation of nociceptive pathways and is considered a reliable method for inducing experimental pain. Additionally, heat stimulation closely mimics certain clinical pain conditions, making it particularly suitable for investigating subjective pain perception and hormonal responses.

Participants were seated comfortably in an armchair in a quiet room maintained at 22−24 °C. Heat stimuli were delivered using the Pathway Thermal Stimulator (Medoc, Ramat Yishai, Israel) [32], with the thermode applied gently to the ventral side of the left forearm at least 30 s before the actual stimulus was delivered. The thermode’s position was changed after each series of heat stimuli to prevent desensitization.

The thermal stimulation pattern, depicted in Figure 1b, began at a baseline temperature of 36 °C and followed a stepwise increase to a peak of 48.5 °C, maintained for 8 s per cycle, with temperature changes occurring every 12 s. The total stimulation lasted 350 s over two cycles. To ensure participant safety, the thermode was repositioned between trials to prevent repeated exposure to the same skin area. Participants were continuously monitored for signs of discomfort or skin irritation throughout the procedure. In addition, emergency measures, such as reducing the stimulation intensity or discontinuing the procedure, were prepared in advance. No adverse effects, including burns or persistent discomfort, were reported during or after the stimulation sessions, confirming the safety and tolerability of the experimental protocol.

Subjective pain intensity during pain stimulation was assessed using a computerized Visual Analogue Scale (VAS), with the left extreme representing no pain and the right extreme representing the worst pain. The participants also responded to the Japanese version of the Short-Form McGill Pain Questionnaire (SF-MPQ-2) to rate the quality of pain after each pain stimulation session. The SF-MPQ-2 includes 22 items across four subscales: continuous pain, intermittent pain, neuropathic pain, and affective descriptors, providing a detailed evaluation of the pain intensity, quality, and emotional impact [33,34].

#### 2.3.2. Hormonal Measurement

Blood samples for hormonal data were collected at five time points (T1–T5) during each session of the experiment:

- T1: before the first heat pain stimulation (baseline),
- T2: after the first heat pain stimulation without a robot hug (Control condition),
- T3: after habituation with the robot and before heat pain stimulation with a robot hug (Robot condition),
- T4: after the second heat pain stimulation with a robot hug (Robot condition),
- T5: at the end, after the Recovery Session (Day 1) or Control Session (Day 2).

While all T1–T4 data were analyzed and presented in the Results, T5 data were excluded from the figures, because they reflected recovery or control conditions and were less relevant to the study’s primary focus on the effects of the robot’s presence during heat pain stimulation. The data from T5 remain available upon request for further context or analysis.

A trained phlebotomist or certified medical professional performed all blood draws using standard aseptic techniques to ensure participant safety and comfort. To minimize discomfort, samples were typically drawn from the same arm unless the participant requested otherwise. Following collection, blood samples were processed to separate serum and plasma, which were then stored at −80 °C until batch analysis to maintain consistency. To ensure sample integrity, blood samples were placed in Protein LoBind Tubes (2.0 mL, Eppendorf, Hamburg, Germany; Catalog No. 022431102), designed to minimize protein loss during storage and handling.

These samples were analyzed for serum levels of cortisol, estradiol, growth hormone (GH), plasma levels of oxytocin (OT), and dehydroepiandrosterone-sulfate (DHEA-S). While this study aimed to assess the estrogen levels, estradiol, a primary and biologically active form of estrogen, was measured as a representative marker. Estradiol is often used in research due to its critical role in regulating physiological processes and its status as the most potent naturally occurring estrogen [35,36,37]. Estradiol analysis was performed exclusively for female participants due to its minimal and less variable presence in males. The use of estradiol as a surrogate for the total estrogen levels aligns with standard practices in endocrinological research. For simplicity and clarity, we will use the term estrogen throughout the text to refer to estradiol.

Hormonal concentrations were determined using enzyme-linked immunosorbent assays (ELISAs) (cortisol: Detect X Cortisol Enzyme Immunoassay Kit, Arbor Assays, Ann Arbor, MI, USA; DHEA-S: DHEA-S ELISA RUO, DRG International, Inc., Springfield, NJ, USA; Estradiol: Estradiol ELISA, ALPCO, Salem, NH, USA; GH: Quantikine ELISA Human Growth Hormone Immunoassay, R&D Systems, Inc., Minneapolis, MN, USA; and OT: Oxytocin ELISA kit, Enzo Life Sciences Inc., Farmingdale, NY, USA). The limit of detection was 45.5 pg/mL (0.00455 ug/dL), 0.044 ug/mL, 10 pg/mL, 2.10 pg/mL, and 15.0 pg/mL for cortisol, DHEA-S, estradiol, GH, and OT, respectively. The intra- and inter-assay coefficients of variation were <10% for all assays, except for OT (<17%).

#### 2.3.3. Mood and Mental Status Assessment Questionnaires

Participants completed the POMS-2, HADS, and Self-Rating Depression Scale (SDS) to evaluate transient mood states, anxiety, and depression.

The Profile of Mood States 2nd Edition (POMS-2) is a standardized psychological questionnaire for assessing transient and distinct mood states (published by Multi-Health Systems Inc., Toronto, ON, Canada) [38,39]. The complete version of the POMS-2 consists of 65 questions and T-scores of six mood clusters as follows: anger hostility (AH), confusion bewilderment (CB), depression dejection (DD), fatigue inertia (FI), tension anxiety (TA), and vigor activity (VA). We determined the total mood disturbance (TMD) by adding the T-scores of AH, CB, DD, FI, and TA (negative mood states) and by subtracting the VA T-score (positive mood state). Friendliness (F) was considered separate from the other mood states.

The Hospital Anxiety and Depression Scale (HADS) comprises two subscales, namely HADS for anxiety (HADS-A) and HADS for depression (HADS-D). Both HADS-A and HADS-D consist of seven items; the participants responded to each item in a four-point (0–3) response category, with possible scores ranging from 0 to 21. Anxiety/depression was defined as HADS-A/HADS-D score ≥8. Anxiety severity was defined as follows: 0−7, no anxiety; 8−10, mild anxiety; 11−14, moderate anxiety; and 15−21, severe anxiety. Depression severity was defined as follows: 0−7, no depression; 8−10, mild depression; 11−14, moderate depression; and 15−21, severe depression [40,41].

Depression was also measured using the Self-Rating Depression Scale (SDS) [42], which includes 20 items constructed based on the clinical diagnostic criteria. The SDS includes positive and negative symptomatic questions (10 each), and each question is scored from 1 to 4 (1 = none or a little of the time, 2 = some of the time, 3 = a good part of the time, and 4 = most or all the time). Depression severity was measured using an index equal to the SDS sum score/80.

### 2.4. Statistical Analyses

Statistical analyses were conducted using JMP^®^, Version 16.0 (SAS Institute Inc., Cary, NC, USA). We performed a paired *t*-test to compare the Control and Robot conditions data. Statistical significance (α) was set at 0.05. Additionally, *p*-values between 0.05 and 0.1 were considered to indicate a trend or tendency toward significance, which may warrant further investigation in future studies with larger sample sizes. While additional significance levels (e.g., ** *p* < 0.01 and *** *p* < 0.005) were used in the figures for descriptive purposes, they are statistical tools to indicate varying levels of evidence against the null hypothesis and do not necessarily imply qualitative differences in the biological or clinical importance of the findings. We performed effect size estimation using Cohen’s *d* test for the paired *t*-test using the formula *d* = Mean_D_/SD_D_, where D = differences of the paired samples values [43]. All data reported in the figures are presented as the means ± SEM.

## 3. Results

### 3.1. Pain Perception Changes Induced by Robot Hug

#### 3.1.1. VAS Results

**Figure 3:**
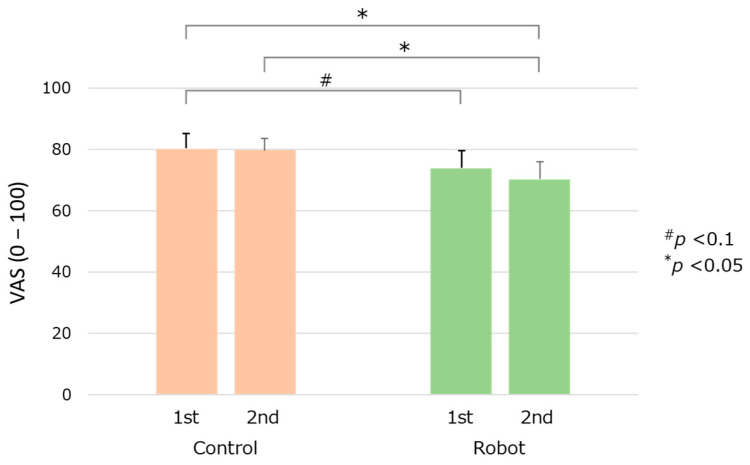
Changes in the subjective pain intensity expressed by VAS. Significant differences are indicated as *# p* < 0.1 or * p < 0.05.

For the Control condition, the maximum pain intensities reported using the computerized VAS were denoted as control_1st and control_2nd for the two peaks of experimental heat pain (Figure 1b) to evaluate the participants’ subjective pain intensity. For the Robot condition, it was expressed as robot_1st and robot_2nd for the two peaks of experimental heat pain. We observed no differences between control_1st and control_2nd (*p* = 0.8203, *d* = 0.04) and between robot_1st and robot_2nd (*p* = 0.4352, *d* = 0.14) (Figure 3). However, robot_2nd subjective pain intensity expressed by the VAS was lower than that of the control_1st and control_2nd conditions (*p* = 0.0307, *d* = 0.39 and *p* = 0.0239, *d* = 0.41, respectively), showing significant small differences.

#### 3.1.2. SF-MPQ-2 Responses

**Figure 4:**
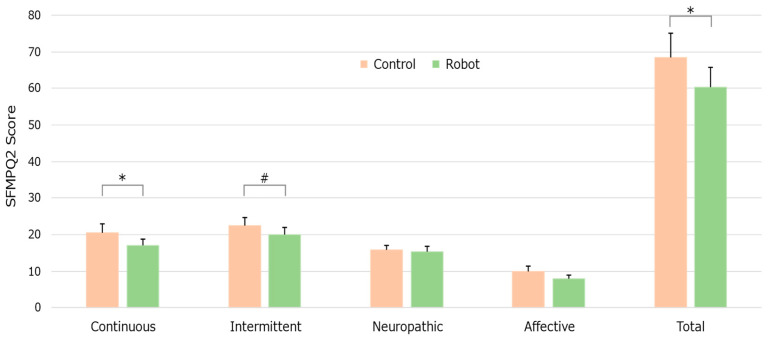
Changes in the pain quality expressed by SFMPQ-2 in the Control and Robot conditions. Significant differences are indicated as # *p* < 0.1 or * *p* < 0.05.

To evaluate the quality of pain, the participants responded to 21 questions about the type of pain experienced using the SF-MPQ-2. The four SF-MPQ-2 subscales (continuous, intermittent, neuropathic, and affective), along with the total score, are visualized in Figure 4. The participants’ subjective expression decreased in the continuous component between the Control and Robot conditions, showing a significant small difference (*p* = 0.011, *d* = 0.48). Furthermore, the total score decreased between the two conditions, showing a significant small difference (*p* = 0.0348, *d* = 0.39). A comparison of self-reported pain intensity levels with and without Moffuly, highlighting significant reductions across each pain type, is shown in Table 2.

### 3.2. Hormone Level Changes

#### 3.2.1. Growth Hormone Levels

**Figure 5:**
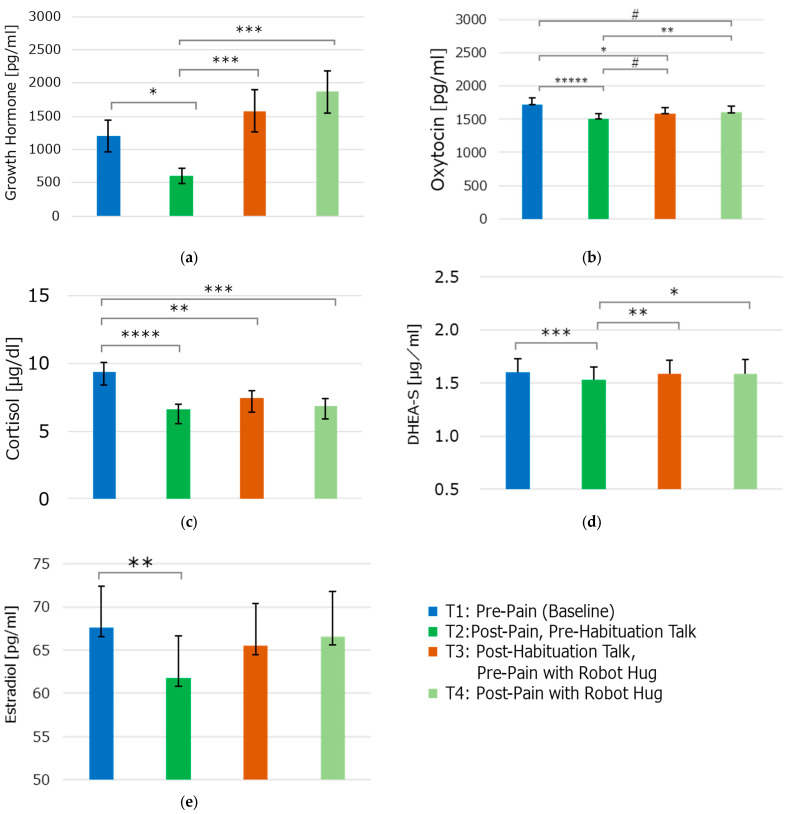
Changes in the serum hormonal levels of (**a**) growth hormone, (**b**) oxytocin, (**c**) cortisol, (**d**) DHEA-S, and (**e**) estrogen (in the form of estradiol) across four time points: T1 (baseline), T2 (post-pain without a robot hug), T3 (post-habituation and pre-pain with a robot hug), and T4 (post-pain with a robot hug). The data from T5 (end of experiment) are excluded, as they reflect recovery or control conditions and were less relevant to the comparisons of the robot’s presence and interaction during the heat pain stimulation. Significant differences are indicated as # *p* < 0.1 (trend toward significance), * *p* < 0.05, ** *p* < 0.01, *** *p* < 0.005, **** *p* < 0.001, and ***** *p* < 0.0005.

The serum GH levels decreased immediately after the experimental heat pain (T2) compared to the baseline (T1). A paired *t*-test and Cohen’s *d* for the paired *t*-test showed a significant medium difference between them (*p* = 0.0113, *d* = 0.46). The GH levels increased after the habituation talk via the robot Moffuly (T3) compared to after the experimental pain (T2) with a significant medium difference (*p* = 0.0043, *d* =0.53), returning to almost baseline levels (*p* = 0.3175, *d* = 0.17 compared to the baseline). Under the Robot condition, the GH level did not decrease after the experimental heat pain (T4) compared to after the habituation talk via a robot (T3, *p* = 0.7662, *d* = 0.05). Comparing the GH levels after the experimental heat pain under the Control and Robot conditions (T2 vs. T4), the Robot condition (T4) showed higher GH levels with a significant medium difference (*p* = 0.0025, *d* = 0.56) (Figure 5a).

#### 3.2.2. Oxytocin Levels

The plasma OT levels decreased immediately after the experimental heat pain (T2) compared to the baseline (T1). The paired *t*-test and Cohen’s *d* for the paired *t*-test showed statistical significance with a large effect size (*p* < 0.0001, *d* = 0.96). The OT slightly increased after the habituation talk via the robot (T3) compared to after the experimental pain (T2, *p* = 0.0829, *d* = 0.31) but did not return to the baseline levels, with a significant small difference (*p* = 0.0327, *d* = 0.38 compared to the baseline). Under the Robot condition, the OT level did not change much after the experimental heat pain (T4) compared to after the habituation talk via robot (T3, *p* = 0.4972, *d* = 0.12). Comparing the OT levels after the experimental heat pain under the Control and Robot conditions (T2 vs. T4), the Robot condition (T4) showed higher OT levels, with a significant small difference (*p* = 0.0079, *d* = 0.49) (Figure 5b).

#### 3.2.3. Cortisol Levels

The serum cortisol levels decreased immediately after the experimental heat pain (T2) compared to the baseline (T1). A paired *t*-test and Cohen’s *d* for the paired *t*-test showed a significant medium difference (*p* = 0.0002, *d* = 0.72). Cortisol slightly increased after the habituation talk via a robot (T3) compared to after the experimental pain (T2, *p* = 0.4285, *d* = 0.14) but did not return to the baseline levels, with a statistically significant medium difference (*p* = 0.0093, *d* = 0.47 compared to the baseline). Under the Robot condition, the cortisol levels slightly decreased after the experimental heat pain (T4) compared to after the habituation talk via a robot (T3, *p* = 0.5342, *d* = 0.11). Comparing the cortisol levels after the experimental heat pain under the Control and Robot conditions (T2 vs. T4), the Robot condition showed slightly higher cortisol levels but with a non-significant small difference (*p* = 0.7387, *d* = 0.058) (Figure 5c).

#### 3.2.4. DHEA-S Levels

The serum DHEA-S levels decreased immediately after the experimental heat pain (T2) compared to the baseline (T1). A paired *t*-test and Cohen’s *d* for the paired *t*-test showed a significant medium difference (*p* = 0.0018, *d* = 0.58). The DHEA-S levels showed a statistically significant increase after the habituation talk via a robot (T3), with a medium effect size compared to after the experimental pain (T2, *p* = 0.0051, *d* = 0.51). Moreover, they showed a similar level to the baseline (*p* = 0.0824, *d* = 0.31 compared to the baseline). Under the Robot condition, the DHEA-S level slightly decreased after the experimental heat pain (T4) compared to after the habituation talk via a robot (T3), with no significant difference (*p* = 0.953, *d* = 0.01). Comparing the DHEA-S levels after the experimental heat pain under the Control and Robot conditions (T2 vs. T4), the Robot condition showed higher levels of DHEA-S, with a significant small difference (*p* = 0.049, *d* = 0.35) (Figure 5d).

#### 3.2.5. Estrogen Levels

The serum estrogen levels decreased immediately after the experimental heat pain (T2) compared to the baseline (T1) (*p* = 0.0125, *d* = 0.53) but not at the other time points (Figure 5e). The differences between the other points were all insignificant, with a very small to small effect size.

#### 3.2.6. Testosterone Levels

The serum testosterone levels did not show significant differences among all the time points, with very small to medium effect sizes.

### 3.3. Mood and Mental Status Responses

#### 3.3.1. POMS-2

**Figure 6:**
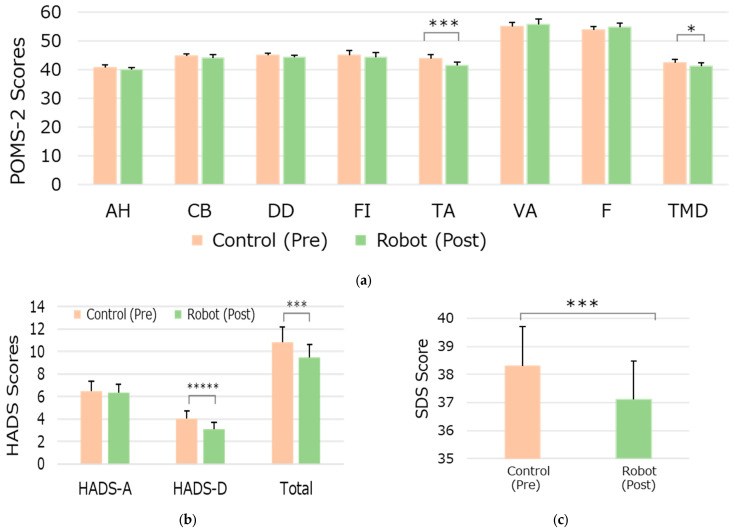
Results of the mood and mental status assessment questionnaires: (**a**) POMS-2, (**b**) HADS, and (**c**) SDS. Significant differences are indicated as * *p* < 0.05, *** *p* < 0.005, and ***** *p* < 0.0005.

Figure 6a depicts the average T-scores of each mood index in the POMS-2 for the Control (pre) and Robot (post) conditions of the experiment’s sessions. The AH, CB, DD, FI, VA, and F scores did not show a significant difference between the Control and Robot conditions. However, the TMD and TA decreased in the Robot condition compared to the Control condition, with significant small or medium differences (*p* = 0.0408, *d* = 0.38 and *p* = 0.0011, *d* = 0.64, respectively).

#### 3.3.2. HADS

The average HADS-Total, HADS-A, and HADS-D scores after the Control (pre) and Robot (post) conditions of the series of experiments are depicted in Figure 6b. The HADS-Total and HADS-D scores decreased after the series of experiments, with significant medium differences (*p* = 0.0040, *d* = 0.55 and *p* = 0.0002, *d* = 0.74, respectively).

#### 3.3.3. SDS

The average SDS scores after the Control (pre) and Robot (post) conditions of the experiment series are shown in Figure 6c. The score decreased after the series of experiments, with a significant medium difference (*p* = 0.0017, d = 0.61).

### 3.4. Recovery Session-Related Results

We observed no significant changes in the hormone levels or questionnaire results, with or without the recovery sessions.

## 4. Discussion

Aging societies face a crucial issue: a shrinking workforce available to provide public services, particularly in healthcare. As the elderly population grows, so does the demand for medical care, putting pressure on an already limited healthcare workforce. This situation calls for exploring innovative approaches to deliver high-quality, cost-effective healthcare, including investments in advanced technologies such as robotics [44].

The healthcare industry has undergone significant changes due to the COVID-19 pandemic, creating both new challenges and opportunities for the robotics sector [45]. Robots have proven their capacity to minimize infection risks and improve care quality. However, their widespread adoption in healthcare settings requires careful evaluation. The function of robots in healthcare will continue to adapt to meet post-pandemic needs, necessitating ongoing assessment and modification to ensure they effectively benefit both patients and the healthcare industry.

In the realm of pain management, robots have demonstrated particular efficacy, especially in pediatric care. They assist with procedures like venipuncture [46,47], help reduce discomfort associated with port access for cancer patients [48,49,50,51], oversee stretching exercises [52], and contribute to the care of elderly individuals [53,54].

The use of interactive care robots for pain relief is a novel application in healthcare. To ensure their effectiveness, these robots should help manage pain by reducing stress, improving mood, providing distractions, and offering social support to prevent isolation and loneliness. Research suggests that social support, even from non-human entities, can mitigate emotional distress and improve pain outcomes [55,56]. Additionally, interactive care robots can complement treatments provided by human caregivers, enhancing quality of life and well-being [13,57].

This study revealed that participants experienced less pain from the experimental heat when the robot Moffuly was present. The observed reduction in VAS scores represents a modest improvement in pain perception. Previous studies suggest that a reduction of 1–2 points on the VAS can be clinically significant in contexts such as acute or chronic pain management [58,59]. While the magnitude of the reduction may appear small, it highlights the potential of robotic interactions to influence subjective pain perception, even in healthy volunteers. These findings align with the hypothesis that addressing loneliness can mitigate emotional distress associated with social isolation, thereby influencing pain perception. This suggests that robotic interactions could serve as a supportive tool in pain management by alleviating loneliness, a known factor in pain exacerbation.

Notably, the POMS-2 test showed significant decreases in T-scores for TA, while the SDS and HADS-D scores also diminished, indicating that robot interactions may help alleviate depressive states. The overall POMS-2 score suggests that the robot had a positive influence on participants’ emotional state and pain perception.

Loneliness is a common issue in mental health disorders, often influencing how individuals perceive and experience pain linked to social isolation. Patients suffering from depression and perceived inadequate social support show poorer outcomes in symptoms, recovery, and social functioning [60]. Furthermore, loneliness is linked to mental health symptoms, stigma, poverty, and a lack of meaningful relationships and belonging [61]. Both loneliness and acute/chronic pain are connected to increased sleep difficulties [62]. Consequently, loneliness and pain are interrelated and can negatively impact mental health and well-being in a two-way relationship [6]. Our experiment involved a 30-min conversation session to familiarize participants with the robot Moffuly. Immediately after this session, the subjects received another series of painful stimuli, with Moffuly serving as a close companion. The robot may have assisted participants in managing their pain by mitigating feelings of loneliness.

The results of this study suggest that the reduction in pain perception may be partially mediated by psychological factors, including reduced depression and perceived loneliness, rather than solely through direct effects on pain processing. Loneliness and pain are closely interrelated, with both contributing to emotional and physiological stress [56,57]. By alleviating feelings of loneliness through robotic interaction, the robot Moffuly likely contributed to a more positive emotional state, which, in turn, influenced participants’ perception of pain. These findings support that addressing emotional and social factors is integral to effective pain management.

Experimental pain stimuli triggered several hormonal changes, including alterations in the GH, OT, cortisol, DHEA-S, and estrogen levels.

These changes varied across hormones and experimental conditions. While most hormones, including GH, OT, cortisol, and DHEA-S, decreased after the first heat pain stimulus (T2), the presence of the robot during the second heat pain stimulus (T4) was associated with attenuated decreases or increases in these hormones compared to the Control condition (T2). For instance, the GH and OT levels were significantly higher in the Robot condition (T4) compared to the Control condition (T2), suggesting a potential modulatory effect of the robot on stress responses. The estrogen levels significantly decreased after the first heat pain stimulus (T2) but showed no significant changes in the subsequent conditions. These findings highlight the variability of hormonal responses and suggest that the robot’s presence may influence stress-related hormonal activity, although the mechanisms remain unclear.

The GH levels significantly decreased following stimulation. GH plays a role in pain modulation and treatment [20], and its related molecules, IGF-1 and ghrelin, influence nociception and chronic pain in both rodents and humans. Low GH and IGF-1 levels are associated with pain hypersensitivity, and GH therapy may benefit patients with fibromyalgia or chronic lower back pain. Childhood psychological aggression is connected to physical health issues such as arthritis and chronic pain [63,64,65]. Despite similar experimental pain intensity, the participants’ subjective pain perception significantly decreased when embraced by the robot Moffuly. GH alterations may reduce pain hypersensitivity, suggesting potential applications for robots in pain therapy.

The experimental heat pain led to reduced OT production, but this effect was not observed when a robot was present. OT exhibits analgesic properties in both animal and human studies and may decrease pain sensitivity by modulating the endogenous opioid system, spinal cord, and brain regions involved in pain processing [23]. Moreover, OT may enhance the positive social and emotional aspects of pain, such as trust, cooperation, and security [66,67]. The pain reduction associated with robotics could be attributed to both hormonal responses and the development of a trusting relationship with the robot.

The adrenal glands release cortisol in response to stress, which has pain-relieving and anti-inflammatory properties. However, long-term stress can disrupt cortisol function, leading to increased pain sensitivity and potentially chronic pain. The Psychoneuroendocrine pathway connects chronic stress, cortisol dysfunction, and pain [68]. Individuals with depression and chronic low back pain show a diminished cortisol response to experimental pain compared to healthy subjects, indicating dysregulation of the hypothalamic–pituitary–adrenal axis [69]. Research suggests that laughter can alter cortisol levels and decrease pain perception in healthy adults, highlighting the role of positive emotions in managing pain [70]. In this study, painful stimulation without robotic intervention resulted in decreased cortisol levels. The presence of Moffuly elicited different cortisol responses to pain, suggesting that the robot induced positive emotions, thus mitigating pain.

DHEA-S, a steroid hormone, plays a role in stress response and pain modulation, potentially offering anti-inflammatory, analgesic, and neuroprotective benefits. Its levels may change based on the frequency and intensity of painful stimuli [71,72]. While the experimental pain significantly affected DHEA-S secretion, this effect was not observed under the robotic conditions, possibly due to a less intense pain experience.

Estrogen impacts pain perception and sensitivity in both males and females. Estrogen receptors, located in pain-related areas such as the spinal cord, thalamus, amygdala, and cerebral cortex, can influence pain-related gene expression, intracellular signaling, neurotransmitter release, and opioid system activity [73,74]. This study revealed that the estrogen levels significantly decreased when subjects experienced pain without robotic assistance.

The endocrine system and brain function are interconnected with DHEA-S, GH, OT, depression, and anxiety. Individuals experiencing depression and anxiety, particularly older adults, often show reduced DHEA-S levels, and supplementing this hormone may alleviate these conditions. DHEA-S impacts neural circuits associated with emotional control and memory, which are compromised in depressive and anxious states [75]. Anxiety and depression are more prevalent in both children and adults with GH deficiency [76], while GH replacement therapy can boost mood, cognitive function, and overall well-being in affected patients [77]. OT, which is affected by psychosocial stress, may serve as a protective factor against anxiety and depression. Administering OT to patients with anxiety disorders can enhance their social abilities, trust, empathy, and positive emotions [78]. The relationship between the endocrine system and mental health is bidirectional, as depression and anxiety can also modify hormone secretion and function.

This study involving a communication robot demonstrated its ability to influence pain perception through reciprocal changes in hormones and mood, indicating potential applications for humanoid robots in pain management. While the results of this study are promising, the practical implementation of such robots in clinical or home settings poses challenges. The cost of developing and deploying humanoid robots with interactive capabilities remains high, and scalability for widespread use is limited by technological and financial constraints. Additionally, patient acceptance and adaptability to robotic interventions can vary, influenced by factors such as cultural attitudes, familiarity with technology, and individual preferences.

Future advancements in robotics could focus on cost-effective designs and modular functionalities tailored to specific healthcare applications. For example, robots could be developed to provide emotional support, distraction, or simple physical comfort during painful procedures or recovery. Expanding the use of these robots beyond pain management to include applications in mental health, elderly care, and rehabilitation could further increase their utility and integration into healthcare systems. Addressing these challenges and diversifying their applications could pave the way for more accessible and practical robotic interventions.

### Limitations and Future Directions

While this study highlights the potential of robots in managing psychological effects and pain perception, several limitations should be acknowledged. First, the study focused on subjective pain perception and hormonal changes rather than direct mechanisms of pain transmission, such as C-fiber activation [79,80]. Future studies should explore the underlying neural and physiological mechanisms of robotic interactions in pain modulation, as well as compare different types of pain stimuli (e.g., mechanical, electrical, or pressure-induced pain) to broaden the applicability of the findings.

Second, the study design cannot fully exclude the possibility of a placebo effect. The placebo effect is known to play a significant role in pain perception and can be influenced by contextual and psychological factors [81]. The observed reduction in pain perception may be influenced by participants’ expectations, the novelty of interacting with a robot, or other psychological factors unrelated to the intervention itself. Future research should incorporate control conditions, such as non-interactive robots or sham interventions, to better isolate the specific effects of robotic interactions on pain perception.

Third, the study only evaluated the immediate effects of robotic interactions on pain perception, mood, and hormonal changes. The long-term persistence of these effects remains unknown. While transient pain reduction has practical applications—such as in post-surgical recovery settings, where a supportive robot could alleviate acute pain and emotional distress—longitudinal studies are needed to examine sustained outcomes.

Fourth, this study was conducted on healthy participants, which may limit the generalizability of the findings to clinical populations experiencing chronic or acute pain. Additionally, the relatively small sample size and lack of blinding may introduce bias or limit the robustness of the findings. Future research should include larger, more diverse samples, implement blinding protocols, and examine cultural factors influencing the effectiveness and acceptance of robotic interventions.

Finally, the hormonal responses were analyzed across a mixed-gender cohort without explicit stratification by gender or age. Hormonal production and effects can vary significantly based on these factors. For example, estrogen (measured as estradiol in this study for female participants) plays a critical role in pain modulation and HPA axis regulation, particularly in women [82]. Similarly, age-related changes in HPA axis reactivity may influence stress and pain responses in older populations [83,84]. Future studies should incorporate stratified analyses to better understand these relationships in diverse cohorts.

The practical challenges of implementing robots in clinical practice must also be addressed. These include evaluating costs, scalability, and patient acceptance and ensuring safety and reliability in diverse healthcare settings. Addressing these challenges will facilitate the broader integration of robots into pain management strategies.

## 5. Conclusions

This study explores the potential of interactive care robots in pain management and emotional support. Interaction with the robot Moffuly significantly reduced subjective pain expression in participants exposed to experimental heat pain. The results also revealed changes in mood indicators, suggesting that robotic interactions may help alleviate emotional distress associated with pain. These effects were accompanied by hormonal changes, including growth hormone, oxytocin, cortisol, DHEA-S, and estrogen, highlighting the interplay between robotic interaction, pain perception, and endocrine function. Although the findings are promising, they should be interpreted with caution due to several limitations, including the small sample size, use of healthy volunteers, and lack of blinding. Future research is needed to validate these findings in clinical populations, explore long-term effects, and further investigate the mechanisms underlying robotic interventions. These results suggest that robotic technologies could complement traditional approaches in pain medicine, anesthesiology, and mental health.

## Figures and Tables

**Table 1:** Questions used in the conversations between participants and Moffuly.

No.	Questions
1	What are you most passionate about right now?
2	What have you enjoyed lately?
3	What do you most want to do, but cannot right now?
4	What do you want right now?
5	If you could know one thing about the future, what would it be?
6	What is your favorite type of person?
7	What do you like to do?
8	What has made you very happy lately?
9	When was the last time you cried? Why did you cry?
10	Tell us what you have done well lately.
11	What is the most precious thing you remember?
12	What is something you have failed at recently?
13	Where would you go on a trip together?
14	If you went to the movies together, what kind of movie would you like to see?
15	What would you like to do for your friend?

**Table 2:** Comparison of the self-reported pain intensity levels with and without Moffuly.

Type of Pain	Control Mean ± SD	With Robot Mean ± SD	*p*-Value
Sharp pain	6.00 ± 0.506	5.41 ± 0.546	0.0778 #
Cramping pain	3.97 ± 0.474	3.03 ± 0.488	0.0494 *
Aching pain	4.18 ± 0.616	3.38 ± 0.562	0.0172 *
Heavy pain	2.45 ± 0.424	1.68 ± 0.358	0.0301 *
Splitting pain	1.94 ± 0.424	1.03 ± 0.233	0.0108 *
Fearful	3.21 ± 0.573	2.29 ± 0.446	0.0416 *
Sharp pain	6.00 ± 0.506	5.41 ± 0.546	0.0778 #
Cramping pain	3.97 ± 0.474	3.03 ± 0.488	0.0494 *
Sharp pain	6.00 ± 0.506	5.41 ± 0.546	0.0778 #

* SD: Standard deviation. Significant differences are indicated as # *p* < 0.1 or * *p* < 0.05.

## Data Availability

The research data supporting the reported results and conclusions of this article will be made available by the corresponding author, without undue reservation.

## Abbreviations

The following abbreviations are used in this manuscript:

AH	Anger hostility
CB	Confusion bewilderment
DD	Depression dejection
DHEA-S	Dehydroepiandrosterone-Sulfate
FI	Fatigue inertia
GH	Growth Hormone
HADS	Hospital Anxiety and Depression Scale
HADS-A	HADS for anxiety
HADS-D	HADS for depression
OT	Oxytocin
POMS-2	Profile of Mood States 2nd Edition
SDS	Self-Rating Depression Scale
SEM	Standard error of the mean
SF-MPQ-2	The Short-Form McGill Pain Questionnaire
TA	Tension anxiety
TMD	Total mood disturbance
VA	Vigor activity
VAS	Visual Analogue Scale
